# Perineural invasion is associated with an aggressive phenotype and reduced *in vitro* capecitabine sensitivity in colorectal cancer

**DOI:** 10.3389/fonc.2026.1961159

**Published:** 2026-10-21

**Authors:** Shuzhe Yang, Zhongyuan Bai, Yanan Li, Chentong Mao, Taohui Han, Shanna Liang, Yanfeng Xi

**Affiliations:** 1 Affiliated Cancer Hospital, Shanxi Medical University, Taiyuan, China; 2 Fifth Clinical Medical School, Shanxi Medical University, Taiyuan, China; 3 Department of Pathology, Shanxi Province Cancer Hospital/Shanxi Hospital Affiliated to Cancer Hosipital, Chinese Academy of Medical Sciences/Cancer Hospital Affiliated to Shanxi Medical University, Taiyuan, China

**Keywords:** capecitabine sensitivity, colorectal cancer, distant metastasis, perineural invasion, tumor microenvironment

## Abstract

**Background:**

Perineural invasion (PNI) is an aggressive biological feature of colorectal cancer (CRC) driven by active crosstalk between tumor cells, Schwann cells, and the neural microenvironment. Although PNI is associated with recurrence, distant metastasis, and chemoresistance, its pathologic diagnosis is constrained by sampling limitations and inter-observer variability, and direct patient-level evidence linking PNI to the cellular sensitivity of specific cytotoxic agents such as capecitabine is lacking.

**Methods:**

We performed a retrospective dual-cohort study of 724 patients with histologically confirmed colorectal adenocarcinoma who underwent R0 resection between January 2024 and August 2025 at a tertiary cancer center in China. Cohort A (n = 430) was used to develop and validate a clinicopathologic nomogram for PNI through LASSO regression, multivariable logistic modeling, bootstrap-corrected internal validation, and internal hold-out validation. Discrimination, calibration, and clinical utility were assessed by area under the receiver operating characteristic curve (AUC), mean absolute error (MAE), Hosmer–Lemeshow test, and decision curve analysis. The association between PNI and distant metastasis was examined in the full cohort by multivariable logistic regression. Cohort B (n = 390) underwent ATP-based *in vitro* tumor chemosensitivity assays on patient-derived tumor cells; the association between PNI status and capecitabine sensitivity was evaluated by ordinal logistic regression with inverse probability of treatment weighting (IPTW) and propensity score matching (PSM).

**Results:**

The six-factor nomogram (T stage, N stage, lymphovascular invasion, tumor budding, KRAS, Ki-67) achieved AUCs of 0.778, 0.741, and 0.803 in the development subset, internal hold-out validation subset and full cohort, respectively, with MAE of 0.023 and Hosmer–Lemeshow P = 0.56. PNI was independently associated with distant metastasis.Patient-derived tumor cells from PNI-positive tumors exhibited markedly reduced *in vitro* capecitabine sensitivity, consistent across IPTW (multivariable OR = 0.62, 95% CI 0.44–0.88, P = 0.007) and PSM (multivariable OR = 0.34, 95% CI 0.15–0.77, P = 0.009) analyses.

**Conclusions:**

PNI-positive CRC harbors a reduced *in vitro* capecitabine sensitivity and an elevated metastatic propensity. The proposed six-factor nomogram offers a practical postoperative tool for PNI risk stratification, while the convergent clinical and *in vitro* evidence may inform hypothesis-generating individualized strategies pending prospective validation of fluoropyrimidine-based adjuvant therapy in PNI-positive patients.

## 1 Introduction

Colorectal cancer (CRC) is among the leading causes of cancer-related incidence and mortality worldwide (1). Despite continuous advancements in multimodal therapies, a considerable proportion of patients still experience disease recurrence and distant metastasis. This clinical reality urgently necessitates the identification of tumor biomarkers with aggressive biological characteristics to guide risk stratification and optimize treatment decision-making.

Among the various aggressive features of CRC, perineural invasion (PNI) has garnered increasing attention. PNI is defined as tumor infiltration of the perineural space, in which tumor cells encircle ≥33% of the nerve circumference or invade the nerve sheat (2). Rather than a passive anatomical event, PNI is an active process driven by crosstalk among cancer cells, Schwann cells, and immune cells through neurotrophic factors, neurotransmitters, and chemokines. This process reduces chemotherapeutic sensitivity by activating pro-survival pathways and establishing an immunosuppressive microenvironment (3). In addition, tumor cells can exploit nerve fibers as migration conduits for regional dissemination, thereby facilitating invasion and metastasis (4, 5). PNI is an independent risk factor for local recurrence, distant metastasis, and poor prognosis in CRC, with a reported incidence of 9–33%. It is also significantly associated with high-risk pathological features, including large tumor size, deep invasion, and lymph node metastasis (6). Therefore, elucidating the mechanisms underlying PNI and its impact on therapeutic response and prognosis is crucial for implementing precision medicine strategies aimed at blocking tumor progression (7).

Despite the established clinical significance of PNI, its routine pathological diagnosis faces substantial challenges. Preoperative biopsy often fails to accurately assess PNI status due to sampling limitations and tumor heterogeneity (8). Even in postoperative specimens, inconsistent diagnostic criteria and inter-observer variability contribute to persistently high rates of PNI underdiagnosis (9, 10). Such limitations frequently leave this critical biological information unavailable when clinicians design surgical plans and adjuvant therapy strategies.

The clinical implications of PNI extend beyond prognostic stratification to encompass treatment response. Currently, capecitabine plus oxaliplatin (XELOX) and its intravenous equivalent FOLFOX-4 are standard regimens for both adjuvant and palliative settings in CRC (11, 12). However, the clinical efficacy of these regimens is modulated by intricate biological mechanisms. Emerging evidence suggests that PNI contributes to chemotherapy resistance through the remodeling of the tumor microenvironment (TME). In transgenic mouse models of pancreatic cancer, both surgical and pharmacological denervation have been shown to significantly enhance chemotherapeutic efficacy, underscoring the inhibitory role of nerves on drug sensitivity. Specifically, nerve-tumor crosstalk can impede chemotherapy response by regulating angiogenesis, inducing immunosuppression, and activating pro-survival signaling pathways (13). Concurrently, studies have confirmed that PNI-positive tumors are closely associated with diminished chemosensitivity in CRC. On one hand, PNI-positive CRC constitutively activates the BDNF/TrkB neurotrophic axis and adrenergic receptor signaling; on the other hand, it fosters an immunosuppressive microenvironment characterized by CD8+ T cell exhaustion and the upregulation of immunosuppressive cytokines (3). However, direct patient-level evidence linking PNI status to sensitivity to specific agents such as capecitabine is still lacking.

Against this backdrop, PNI is widely recognized as an established adverse prognostic factor in CRC, and its clinical importance has reached a consensus. However, pathological assessment of PNI remains constrained by certain limitations, including insufficient tissue sampling, inter-observer variability, and missing PNI data in some cases. Furthermore, the impact of PNI status on the efficacy of specific chemotherapy regimens requires further investigation. This study therefore aimed to (1) develop and validate a clinicopathologic nomogram for predicting PNI from routinely available clinical parameters, and (2) assess the impact of PNI status on distant metastasis and *in vitro* capecitabine sensitivity to inform individualized adjuvant therapy.

## 2 Materials and methods

### 2.1 Patient selection and follow-up

This single-center retrospective cohort study enrolled patients with histologically confirmed colorectal adenocarcinoma who underwent R0 resection between January 2024 and August 2025. The follow-up duration in this study ranged from 8 to 27 months, with a median of 18 months. The shortest follow-up was 8 months and the longest was 27 months. Based on the enrollment period and the date of data retrieval, the estimated mean follow-up was approximately 17.5 months. Variation in follow-up duration may affect the detection of distant metastasis, as patients with shorter follow-up have less opportunity to develop distant metastasis, which could attenuate the association between PNI and distant metastasis. We evaluated this influence through a sensitivity analysis restricted to patients with at least 12 months of follow-up, and the direction of the association remained consistent, suggesting that the variation in follow-up duration did not substantially affect the primary findings. The exclusion criteria were: coexistence of other primary malignancies, receipt of neoadjuvant chemoradiotherapy prior to surgery, and hereditary CRC syndromes. Postoperative follow-up was extended through March 2026, and the database was locked on March 31, 2026 for the present analysis. A total of 724 patients were included (**Figure 1**). This study was approved by the Medical Ethics Committee of Shanxi Provincial Cancer Hospital (No. 202112), and all patients provided written informed consent. The study was conducted in accordance with the Declaration of Helsinki.

**Figure 1:**
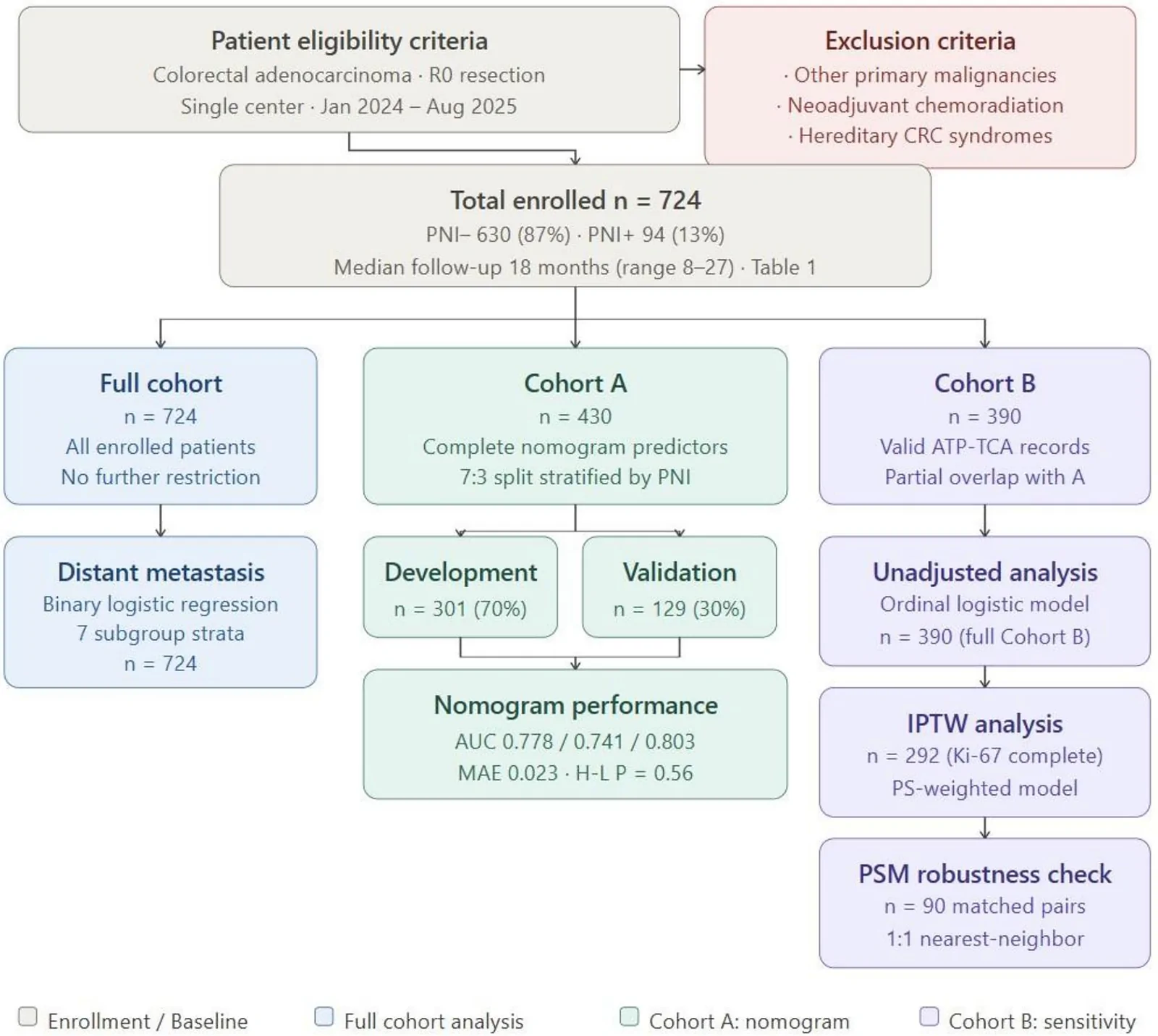
Study flowchart detailing patient selection and cohort derivation. A total of 724 patients with colorectal adenocarcinoma were included. Cohort A (n = 430) was split into development and validation subsets for nomogram construction. Cohort B (n = 390) was utilized for capecitabine sensitivity analysis using unadjusted, weighted, and matched models. PNI, perineural invasion; CRC, colorectal cancer; AUC, area under the receiver operating characteristic curve; MAE, mean absolute error; H-L, Hosmer-Lemeshow; IPTW, inverse probability of treatment weighting; PS, propensity score; PSM, propensity score matching.

All patients were prospectively followed from the date of surgery to the date of confirmed distant metastasis, death, or last contact, whichever occurred first. However, exact dates of distant metastasis were not systematically captured as structured variables. Dates in routine clinical records often reflect reporting, testing, or treatment dates rather than the true date of metastasis, and a reliable time zero, event time, and censoring process could not be reconstructed retrospectively. Therefore, time-to-event analysis was not used as the primary analysis. Follow-up consisted of outpatient visits every 3 months during the first postoperative year and every 6 months thereafter, supplemented by telephone interviews when in-person visits were unavailable. Distant metastasis was confirmed by contrast-enhanced computed tomography, magnetic resonance imaging, or 18F-FDG positron emission tomography–computed tomography, with histopathologic confirmation when clinically feasible. Patients lost to follow-up were censored at the date of last documented contact.

### 2.2 Pathological assessment and variable definitions

PNI was defined according to the Liebig criteria as tumor cell infiltration encircling ≥33% of the nerve circumference or involving any layer of the nerve sheath. Hematoxylin and eosin (H&E) slides were independently reviewed by two senior gastrointestinal pathologists who were blinded to clinical outcomes.

Tumor budding was graded within a 0.785 mm^2^ hotspot field according to the 2016 International Tumor Budding Consensus Conference (ITBCC) guidelines: Bd1 (0–4 buds), Bd2 (5–9 buds), and Bd3 (≥10 buds). Ki-67 expression was evaluated via immunohistochemistry and dichotomized into high (≥50%) and low (<50%) expression based on the cohort median. KRAS mutation status (exons 2, 3, and 4) was detected using amplification refractory mutation system PCR (ARMS-PCR). Distant metastasis was defined as radiologically or histologically confirmed tumor spread to non-regional organs (e.g., liver, lung, peritoneum, distant lymph nodes, or bone) during the follow-up period.

### 2.3 *In vitro* chemosensitivity assay

For patients with available records of *in vitro* chemosensitivity testing, fresh tumor tissues were harvested immediately following surgical resection. Single-cell suspensions were prepared using mechanical and enzymatic methods, and chemosensitivity was evaluated using the adenosine triphosphate-based bioluminescence assay for tumor chemosensitivity (ATP-TCA). Following seeding into 96-well plates, cells were exposed to the test drugs at six gradient concentrations (200%, 100%, 50%, 25%, 12.5%, and 6.25% of the theoretical drug concentration [TDC]) for 72 hours. TDC was defined as the peak plasma concentration PPC achieved with standard-dose capecitabine 1250 mg per square meter twice daily. Intracellular ATP levels were quantified via a luciferase-based luminescence assay to calculate the tumor growth inhibition rate (TGI):


$$\text{TGI}(\%)=(1-\frac{\left(\text{Test}-\text{MI}\right)}{\left(\text{M}0-\text{ MI}\right)})\times 100\%$$

Where Test, M0, and MI represent the ATP luminescence values of the drug-treated group, negative control group, and maximum inhibition control group, respectively. Sensitivity was classified into four grades based on the IC_50_, IC_90_, and the comprehensive inhibition index (Index): Grade 3 (sensitive), IC_50_ ≤ 25% TDC and IC_90_ ≤ 100% TDC; Grade 2 (moderately sensitive), IC_50_ ≤ 25% TDC and IC_90_ > 100% TDC; Grade 1 (mildly sensitive), IC_50_ > 25% TDC and IC_90_ < 100% TDC, or Index < 300; Grade 0 (resistant), Index ≥ 300. For quality control, a sample’s test results were considered valid only if the ATP luminescence value of the negative control wells (M0) exceeded 5000 RLU (relative light units) and the coefficient of variation (CV) was less than 20%; otherwise, the sample was excluded.

The tested drugs encompassed fluorouracil (5-FU), capecitabine, and oxaliplatin. Given the central role of capecitabine as the cornerstone oral fluoropyrimidine in CRC adjuvant therapy and its mechanistic susceptibility to nerve–tumor crosstalk, capecitabine sensitivity was pre-specified as the primary outcome of Cohort B, whereas 5-FU and oxaliplatin sensitivities were treated as exploratory secondary outcomes. No exogenous activation system was added. Previous studies have shown that colorectal cancer cells can express the enzymes like thymidine phosphorylase required for capecitabine activation and can generate 5-FU locally.

### 2.4 Cohort design

This study adopted a dual-cohort design (**Figure 1**). The 724 enrolled patients constituted the baseline description population (**Table 1**), from which two analytically independent cohorts were derived. Cohort A (n = 430), comprising patients with complete data for all candidate nomogram predictors, was used for development and validation of the PNI prediction model. Cohort B (n = 390), comprising patients with valid *in vitro* capecitabine sensitivity records, was used for the chemosensitivity association analysis. Although the two cohorts partially overlapped at the patient level, model training and chemosensitivity testing were performed separately to prevent information leakage.

**Table 1:** Baseline characteristics of 724 patients with colorectal cancer stratified by perineural invasion status.

Characteristic	Overall N = 724*^1^*	PNI (−) N = 630*^1^*	PNI(+) N = 94*^1^*	p-value*^2^*
Gender				0.7
Female	303 (42%)	262 (42%)	41 (44%)	
Male	421 (58%)	368 (58%)	53 (56%)	
Age				0.3
≤65 years	366 (51%)	314 (50%)	52 (55%)	
>65 years	358 (49%)	316 (50%)	42 (45%)	
Tumor Location				0.2
Colon	350 (48%)	311 (49%)	39 (41%)	
Rectum	374 (52%)	319 (51%)	55 (59%)	
Depth of Invasion (pT)				0.003*
T1	7 (1.0%)	6 (1.0%)	1 (1.1%)	
T2	146 (20%)	139 (22%)	7 (7.4%)	
T3	549 (76%)	465 (74%)	84 (89%)	
T4	22 (3.0%)	20 (3.2%)	2 (2.1%)	
Tumor Budding Grade				<0.001*
BD1	290 (48%)	274 (52%)	16 (21%)	
BD2	95 (16%)	78 (15%)	17 (22%)	
BD3	224 (37%)	179 (34%)	45 (58%)	
Missing	115	99	16	
Lymphovascular Invasion				<0.001*
Absent	561 (77%)	509 (81%)	52 (55%)	
Present	163 (23%)	121 (19%)	42 (45%)	
Lymph Node Metastasis (pN)				<0.001*
N0	444 (61%)	409 (65%)	35 (37%)	
N1	191 (26%)	155 (25%)	36 (38%)	
N2	89 (12%)	66 (10%)	23 (24%)	
KRAS Mutation Status				0.014*
Wild-type	365 (51%)	333 (53%)	32 (39%)	
Mutant	348 (49%)	297 (47%)	51 (61%)	
Missing	11	0	11	
NRAS Mutation Status				0.8
Wild-type	681 (96%)	602 (96%)	79 (95%)	
Mutant	32 (4.5%)	28 (4.4%)	4 (4.8%)	
Missing	11	0	0	
BRAF Mutation Status				0.5
Wild-type	690 (97%)	608 (97%)	82 (99%)	
Mutant	23 (3.2%)	22 (3.5%)	1 (1.2%)	
Missing	11	0	0	
Microsatellite Status				0.044*
MSS/MSI-L	536 (93%)	462 (92%)	74 (99%)	
MSI-H	39 (6.8%)	38 (7.6%)	1 (1.3%)	
Missing	149	130	19	
Ki-67 Expression				0.013*
≤50%	25 (4.9%)	17 (3.9%)	8 (11%)	
>50%	486 (95%)	424 (96%)	62 (89%)	
Missing	213	189	24	
P53 Expression				0.2
Negative	137 (26%)	123 (27%)	14 (20%)	
Overexpression	106 (20%)	94 (21%)	12 (17%)	
Null (Complete Loss)	277 (53%)	233 (52%)	44 (63%)	
Missing	204	180	24	
HER2 Expression				0.2
0	290 (54%)	251 (54%)	39 (54%)	
1+	191 (36%)	161 (35%)	30 (42%)	
2+	44 (8.3%)	42 (9.1%)	2 (2.8%)	
3+	8 (1.5%)	7 (1.5%)	1 (1.4%)	
Missing	191	169	22	
NLR Expression				0.6
Low	256 (36%)	220 (35%)	36 (38%)	
High	462 (64%)	404 (65%)	58 (62%)	
Missing	6	6	0	
LMR Expression				>0.9
Low	97 (14%)	84 (13%)	13 (14%)	
High	621 (86%)	540 (87%)	81 (86%)	
Missing	6	6	0	

^1^n (%) ^2^Pearson’s Chi-squared test; Fisher’s exact test.

CRC, colorectal cancer; PNI, perineural invasion; pT, depth of invasion; BD, tumor budding; pN, lymph node metastasis; NLR, neutrophil-to-lymphocyte ratio; LMR, lymphocyte-to-monocyte ratio.

Cohort A was randomly split into a development subset (n = 301) and an internal hold-out validation subset (n = 129) at a 7:3 ratio, stratified by PNI status to preserve outcome distribution. The validation subset was entirely withheld from variable selection and model fitting. In Cohort B, univariate analyses were conducted on the full sample (n = 390), whereas multivariable models adjusting for Ki-67 were restricted to patients with complete Ki-67 data (n = 292) so as to preserve statistical power in the unadjusted analyses.

### 2.5 Construction of the PNI prediction model

We pre-specified 17 candidate clinicopathological variables based on their established prognostic significance in colorectal cancer and their documented association with PNI. Variable selection was performed using 10-fold cross-validated LASSO logistic regression (R package glmnet, family = “binomial”, alpha = 1, type.measure = “auc”), with lambda.min selected as the optimal penalty parameter to maximize statistical power. Subsequently, core independent predictors were identified by combining univariate logistic regression (P < 0.05) with backward stepwise selection based on the Akaike Information Criterion (AIC). The neutrophil-to-lymphocyte ratio (NLR) and lymphocyte-to-monocyte ratio (LMR) failed to reach statistical significance in both univariate and multivariate models, and their coefficients were compressed to near zero in the LASSO regression, suggesting that their associations with PNI might be attributable to confounding effects from collinear variables; thus, they were excluded from the final model. Although Ki-67 exhibited a relatively large negative coefficient in the LASSO regression, it was retained considering its biological relevance to tumor dedifferentiation. Ultimately, the final nomogram integrated the stability of LASSO screening, statistical evidence, and clinicopathological rationale. Based on clinical consensus, we added T stage and LVI to form the six-factor model, as both are NCCN/AJCC-recommended, biologically linked to PNI, and may have been attenuated by collinearity with pN. This integration reduces overfitting while enhancing clinical utility. The overall explanatory power of the model was quantified using Nagelkerke’s R^2^, and the visual nomogram was constructed using the rms package.

### 2.6 Validation of the PNI prediction model

Model performance was evaluated using a dual strategy of bootstrap internal validation and internal hold-out validation. Discriminatory ability was quantified by the area under the receiver operating characteristic curve (AUC) with 95% confidence intervals. For the development subset (n = 301), optimism-corrected AUC was estimated by bootstrap resampling (2,000 iterations, R package pROC) to control for overfitting. For the internal hold-out validation subset (n = 129) and the full Cohort A (n = 430), apparent AUC and DeLong-based 95% CIs were reported. A difference in AUC between development and validation subsets of less than 0.05 was pre-specified as the threshold for acceptable generalizability. Calibration was assessed using two complementary approaches: for the full cohort (n= 430), bias-corrected calibration curves based on bootstrap resampling (1000 resamples, R package rms) were generated, and the mean absolute error was used to quantify calibration deviation. For the independent validation set (n= 129), a grouped calibration plot combined with the Hosmer–Lemeshow test (10 groups) was applied, with P> 0.05 indicating no significant lack of fit. Clinical utility was evaluated using decision curve analysis (DCA), which calculated the net benefit across the modeling subset, validation subset, and full cohort, comparing the model against the “treat all” and “treat none” strategies.

### 2.7 Association analysis between PNI and distant metastasis

Using the full cohort (n=724), distant metastasis (DM) served as the outcome variable, and binary logistic regression was employed to evaluate the association between PNI and DM. The primary endpoint was the occurrence of distant metastasis during the follow-up period, defined by structured pathological, radiological, and follow-up records. Because exact event times and censoring times could not be reliably constructed, binary logistic regression was used to estimate an association rather than a hazard. Firth penalized logistic regression and E-value analysis were performed as sensitivity analyses. Candidate variables for univariate analysis included PNI, age, sex, tumor location, pT, pN, LVI, KRAS, Ki-67, and TB. Variables with a univariate P < 0.10 were entered into the multivariate model. Effect sizes are presented as odds ratios (ORs) with 95% confidence intervals (CIs), with statistical significance set at P < 0.05. Furthermore, stratified subgroup analyses were conducted according to age, sex, tumor location, T stage, N stage, LVI, and KRAS status to assess the stability of the association.

### 2.8 Association analysis between PNI status and capecitabine sensitivity

Cohort B (n = 390) was utilized to evaluate the association between PNI status and capecitabine sensitivity. Capecitabine sensitivity grade (0–3, an ordinal variable) served as the primary outcome, with PNI as the main exposure factor. An ordinal logistic regression model was applied, adjusting for age, sex, tumor location, pT, pN, LVI, KRAS, and Ki-67. The proportional odds assumption was formally tested using the Brant test and an approximate likelihood-ratio test. A partial proportional odds model and multinomial logistic regression were also performed as sensitivity analyses. To further control for confounding, inverse probability of treatment weighting (IPTW) was implemented. Propensity scores were estimated via logistic regression based on the aforementioned covariates, with weights truncated at the 99th percentile. Covariate balance was assessed using the standardized mean difference (SMD). Robustness was evaluated using propensity score matching (PSM) with nearest-neighbor 1:1 matching (caliper width = 0.25 SD), matching on pT, pN, LVI, tumor location, and KRAS. Covariate balance before and after weighting and matching was assessed using absolute standardized mean differences ASMD, with ASMD less than 0.1 considered adequate balance.

To minimize selection bias, all consecutive eligible patients during the study period were enrolled. Information bias was reduced through blinded pathological review and standardized molecular assays. Confounding bias was addressed using multivariable ordinal logistic regression, inverse probability of treatment weighting (IPTW), and propensity score matching (PSM). The final cohort comprised all available eligible cases, ensuring representative real-world data. The robustness of the findings was strengthened by the consistency of results across three complementary statistical approaches (unadjusted, IPTW, and PSM models), which all indicated a significant association between PNI and reduced capecitabine sensitivity.

### 2.9 Statistical analysis

All statistical analyses were performed using R software (version 4.5.3), relying primarily on the following packages: glmnet, rms, pROC, MASS, WeightIt, MatchIt, cobalt, caret, and ggplot2. Continuous variables are described as means ± standard deviations or medians (interquartile ranges), while categorical variables are presented as frequencies and percentages. Group comparisons were conducted using Student’s t-test or Mann–Whitney U test for continuous variables, and chi-square test or Fisher’s exact test for categorical variables. Missing data were summarized using the naniar package. Complete-case analysis was used for the primary model. Sensitivity analyses included missing-indicator models, multiple imputation, worst-case scenario analysis for Ki-67, and Firth penalized logistic regression. E-value was calculated using the VanderWeele and Ding method. All tests were two-sided, with a significance threshold set at P < 0.05. The random seed was uniformly set to 2024.

## 3 Results

### 3.1 Baseline characteristics and cohort description

Baseline characteristics were analyzed for the total cohort of 724 patients. Among them, 630 were PNI-negative and 94 were PNI-positive. The two groups were comparable in sex, age, and tumor location. Due to varying numbers of missing values across Tumor Budding Grade, KRAS mutation status, Ki-67 expression, NLR, and LMR, we conducted the analysis on a complete-case basis. The PNI-positive group exhibited more aggressive pathological features: the proportion of pT3 stage was significantly higher, and the rates of lymphatic vascular invasion (LVI), lymph node metastasis, and high-grade tumor budding (BD3) were all markedly elevated. The KRAS mutation rate was significantly higher in PNI-positive tumors. Notably, the proportion of high Ki-67 expression was lower in PNI-positive than PNI-negative tumors. No significant differences were observed in NLR (P = 0.6) or LMR (P > 0.9). Detailed comparisons are provided in **Table 1**. Among the 724 patients, 430 were included in the complete-case analysis and 294 were excluded for missing modeling variables. Age P = 0.416, sex P = 0.641, and PNI status P = 0.969 were balanced between groups. Tumor location P = 0.036 and pT stage P = 0.012 differed. pN stage P = 0.068 was borderline. PNI as the exposure of interest was completely balanced. All models were analyzed using complete-data subsets. Multiple imputation for the PNI prediction model yielded ORs consistent with the complete-case analysis. The distant metastasis model included PNI, LVI, and pN with no missing values. The complete-covariate subset for the IPTW and PSM analyses of capecitabine sensitivity had n = 292 with no missing values. Multiple imputation and missing-indicator analyses for variables with missingness yielded consistent directions, supporting the robustness of the findings.

### 3.2 Six-factor PNI prediction nomogram

The optimal penalty parameter for LASSO regression was λ_min = 0.00076 (**Figures 2A, B**).These six variables were prioritized as the dominant positive predictive signals. All 17 candidate variables were subsequently entered into univariate logistic regression for confirmation. At λ_min, T stage, N stage, KRAS, tumor location, LVI and tumor budding showed the strongest positive coefficients, while Ki-67 exhibited the largest negative coefficient (**Figure 2C**). The six-factor model achieved an AUC of 0.803, compared with 0.754 for the four-factor model in the same cohort, supporting the incremental value of T stage and LVI. These six positive predictors were retained as the dominant predictive signals for subsequent modeling. Univariate and multivariate logistic regression analyses identified tumor budding, KRAS, pN stage, and Ki-67 as independent predictors (**Figures 2D, E**). Integrating LASSO stability, statistical evidence, and clinical plausibility, we supplemented the four core factors with LVI and T stage—two clinically recognized indicators—to construct a six-factor extended prediction model and generate the nomogram (**Figure 2F**).

**Figure 2:**
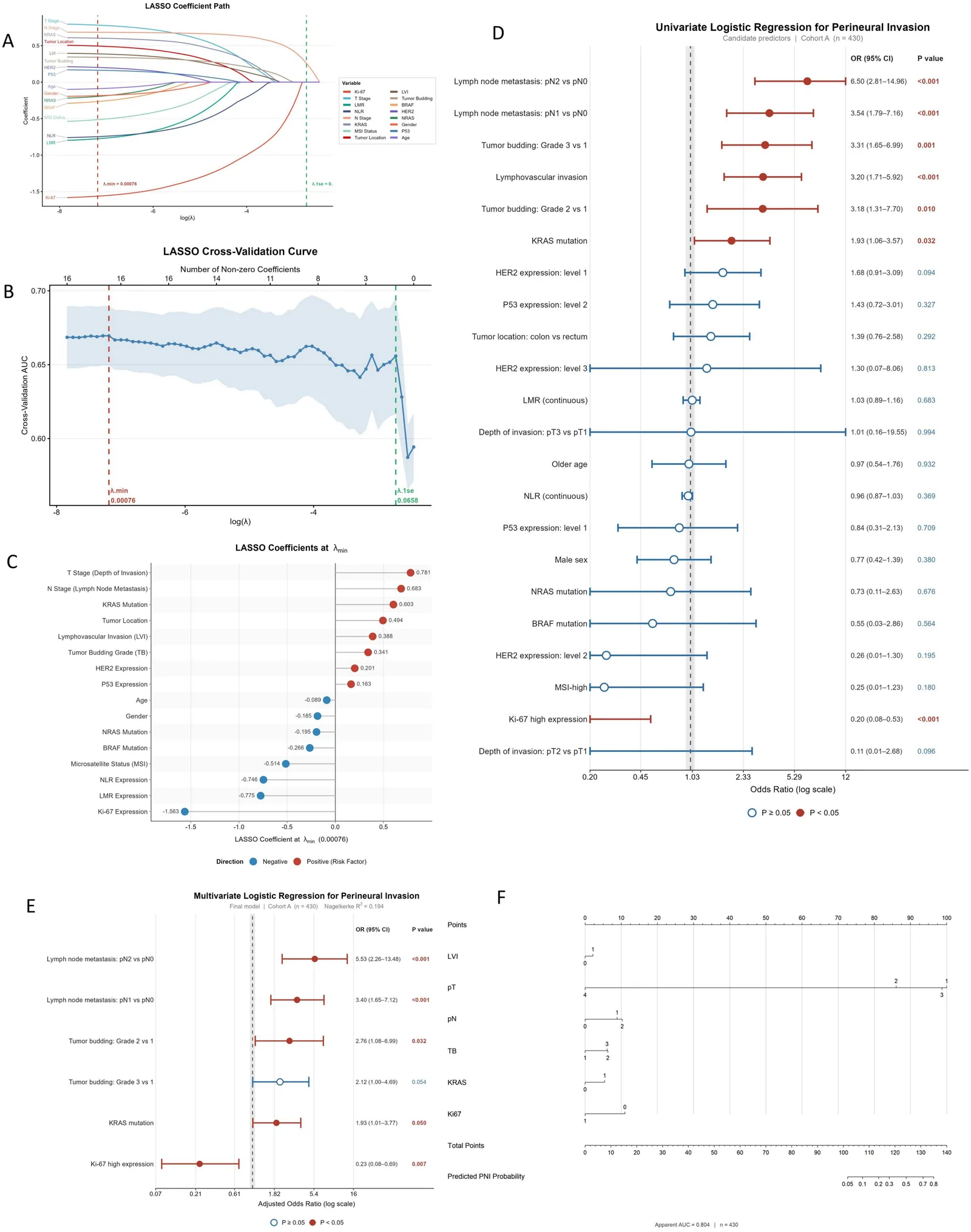
Construction of the perineural invasion prediction nomogram. **(A, B)** Least absolute shrinkage and selection operator (LASSO) coefficient profiles and cross-validation curve identifying optimal penalization. **(C)** Core predictors selected at the optimal lambda. **(D, E)** Univariate and multivariable logistic regression identifying independent predictors. **(F)** The final six-factor predictive nomogram incorporating T stage and lymphovascular invasion. LVI, lymphovascular invasion; pT, depth of invasion; pN, lymph node metastasis; TB, tumor budding; OR, odds ratio; CI, confidence interval.

### 3.3 Validation and performance comparison of the PNI prediction model

The six-factor model demonstrated superior discriminative ability compared to the four-factor reduced model. The AUCs for the modeling subset, internal hold-out validation subset, and entire cohort were 0.778, 0.741, and 0.803 respectively (**Figures 3A–C**). The difference in AUC between the training and validation sets was 0.037, which was below the predefined threshold. The apparent AUC of the four-factor reduced model was 0.754, and the bootstrap-corrected AUC was 0.730, both lower than those of the six-factor model (**Figure 3D**). Calibration curves indicated good agreement between predicted probabilities and actual observed probabilities (**Figures 3E, F**). The Hosmer–Lemeshow test for the internal hold-out validation subset showed no significant lack of fit (P > 0.05), suggesting acceptable discrimination and calibration performance. Decision curve analysis (DCA) revealed that the six-factor nomogram yielded a positive net benefit across threshold probabilities of approximately 5% to 40%, with consistent clinical utility yielding higher net benefit than the ‘treat-all’ and ‘treat-none’ reference strategies (**Figures 3G–I**). These results confirm that guiding the screening and intervention for high-risk PNI patients based on the model’s predicted probabilities holds substantial potential for clinical benefit.

**Figure 3:**
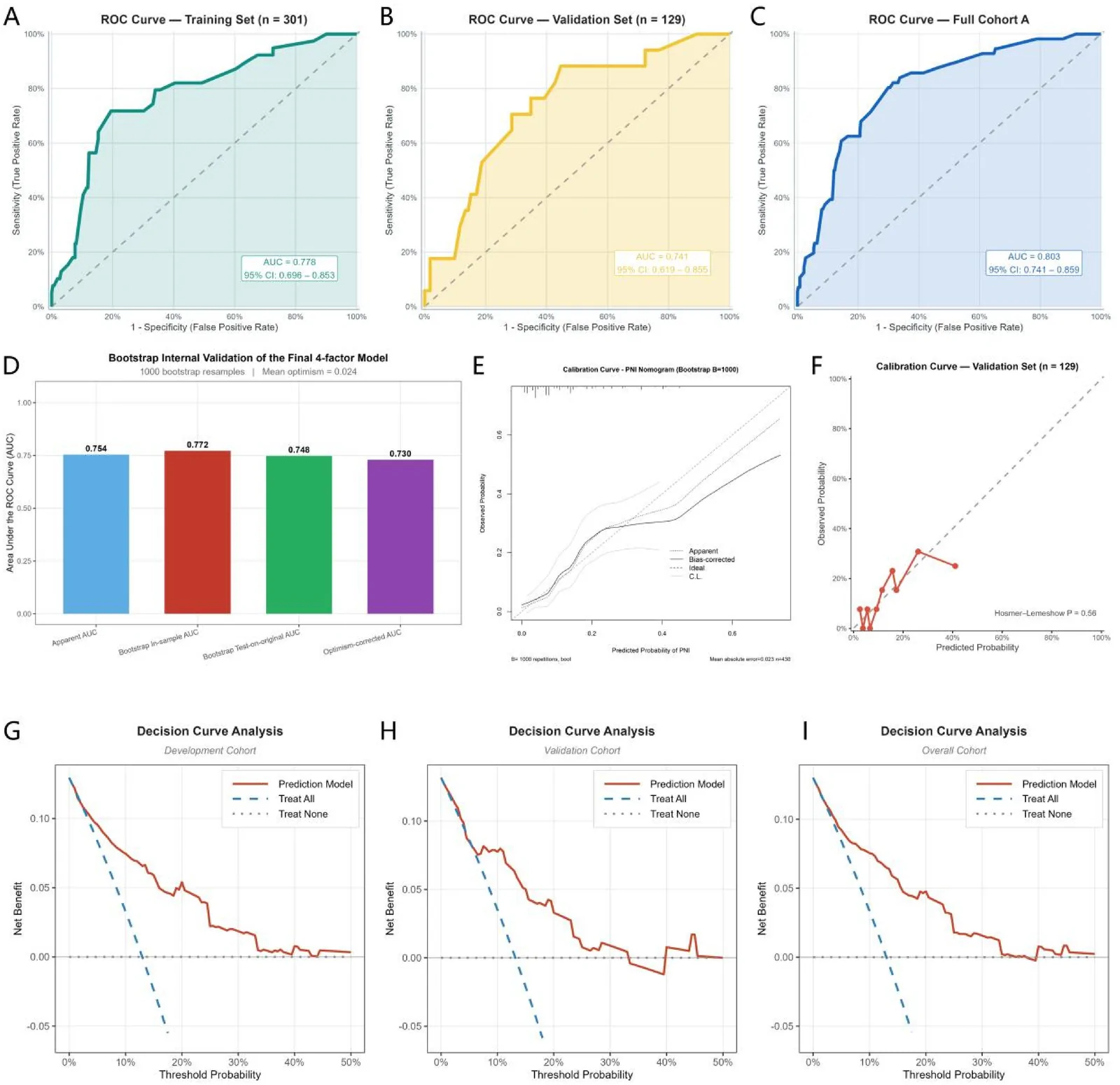
Performance evaluation of the prediction model. **(A–C)** Receiver operating characteristic curves demonstrating discriminative ability in the training, validation, and full cohorts. **(D)** Bootstrap internal validation of the reduced model. **(E, F)** Calibration curves showing agreement between predicted and observed probabilities. **(G–I)** Decision curve analysis indicating consistent net clinical benefit across varying threshold probabilities. AUC, area under the receiver operating characteristic curve; CI, confidence interval; C.L., confidence limit; PNI, perineural invasion.

### 3.4 PNI is independently associated with distant metastasis

Complete follow-up data were available for all 724 patients until March 2026. The follow-up duration ranged from 8 to 27 months, with a median of 18 months. Metastasis occurred in 84 patients. Univariate logistic regression showed a significant association between PNI and distant metastasis (OR = 2.80, 95% CI: 1.60–4.77, P < 0.001). After incorporating PNI, LVI, and pN into the multivariate model, PNI remained independently associated with distant metastasis.(OR = 2.38, 95% CI: 1.32–4.17, P = 0.003; **Figure 4A**). Firth penalized logistic regression yielded results virtually identical to the primary analysis, with PNI OR of 2.38, 95% CI 1.33 to 4.14, and P = 0.004. The E-value was 3.61 with a lower confidence limit of 1.88, calculated using the VanderWeele and Ding 2017 method with the common-outcome approximation. The baseline risk in PNI-negative patients was 9.84%, which is 62/630. This indicates that an unmeasured confounder would need to be associated with both PNI and distant metastasis by a risk ratio of at least 3.61-fold to fully explain away the observed association. Subgroup analyses showed that PNI-associated increase in distant metastasis risk was directionally consistent and statistically significant across subgroups stratified by age, sex, tumor location, T3 stage, N2 stage, LVI status, and KRAS wild-type status. No significant associations were observed in the subgroups of T2, N0, N1, or KRAS mutant status. However, the PNI and KRAS interaction was not statistically significant (LR chi-square = 2.60, df = 1, P = 0.107). The association was directionally stronger in KRAS wild-type tumors (OR = 4.61, 95% CI 1.99–10.24) than in KRAS-mutant tumors (OR = 1.53, 95% CI 0.59–3.54). This subgroup finding should be considered exploratory. Overall, these results indicate cross-subgroup stability of the association (**Figure 4B**).

**Figure 4:**
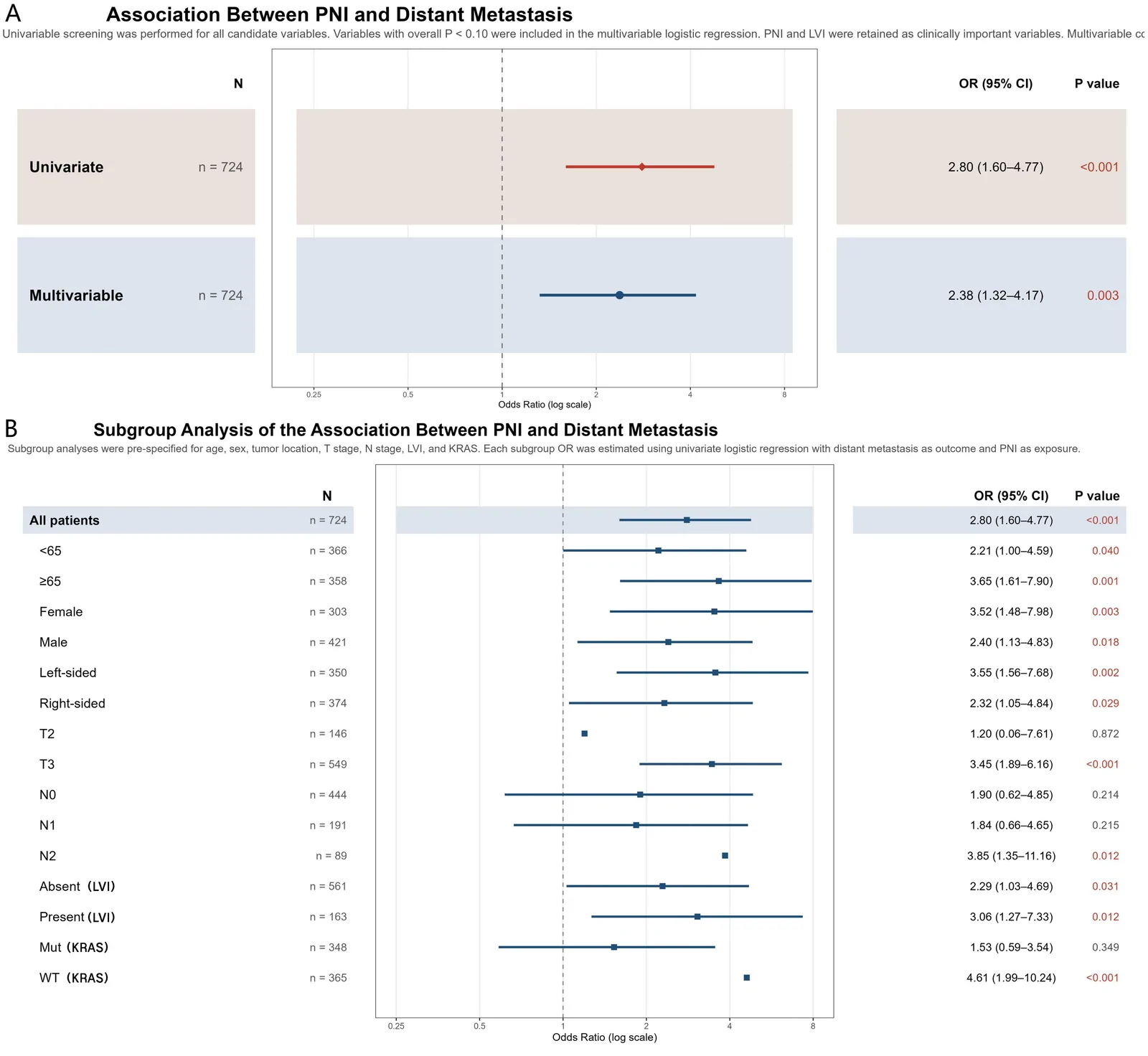
Association between perineural invasion and distant metastasis. **(A)** Univariable and multivariable logistic regression showing that perineural invasion was independently associated with distant metastasis in the full cohort(n = 724). **(B)** Subgroup analyses confirming the consistency of this association across various clinicopathologic strata, particularly in patients with wild-type KRAS. PNI, perineural invasion; OR, odds ratio; CI, confidence interval; LVI, lymphovascular invasion; Mut, mutant; WT, wild-type.

### 3.5 Association between PNI status and *in vitro* capecitabine sensitivity

The distribution of capecitabine sensitivity grades differed substantially between PNI groups (**Figure 5A**). In PNI-negative tumors, 12.6% were classified as Grade 3 (high sensitivity), 27.3% as Grade 2 (moderate), 51.0% as Grade 1 (low), and 9.1% as Grade 0 (resistant). In PNI-positive tumors, the resistant fraction (Grade 0) rose markedly to 22.4%, while Grade 3 decreased to 10.2%, suggesting a rightward shift toward chemoresistance.

**Figure 5:**
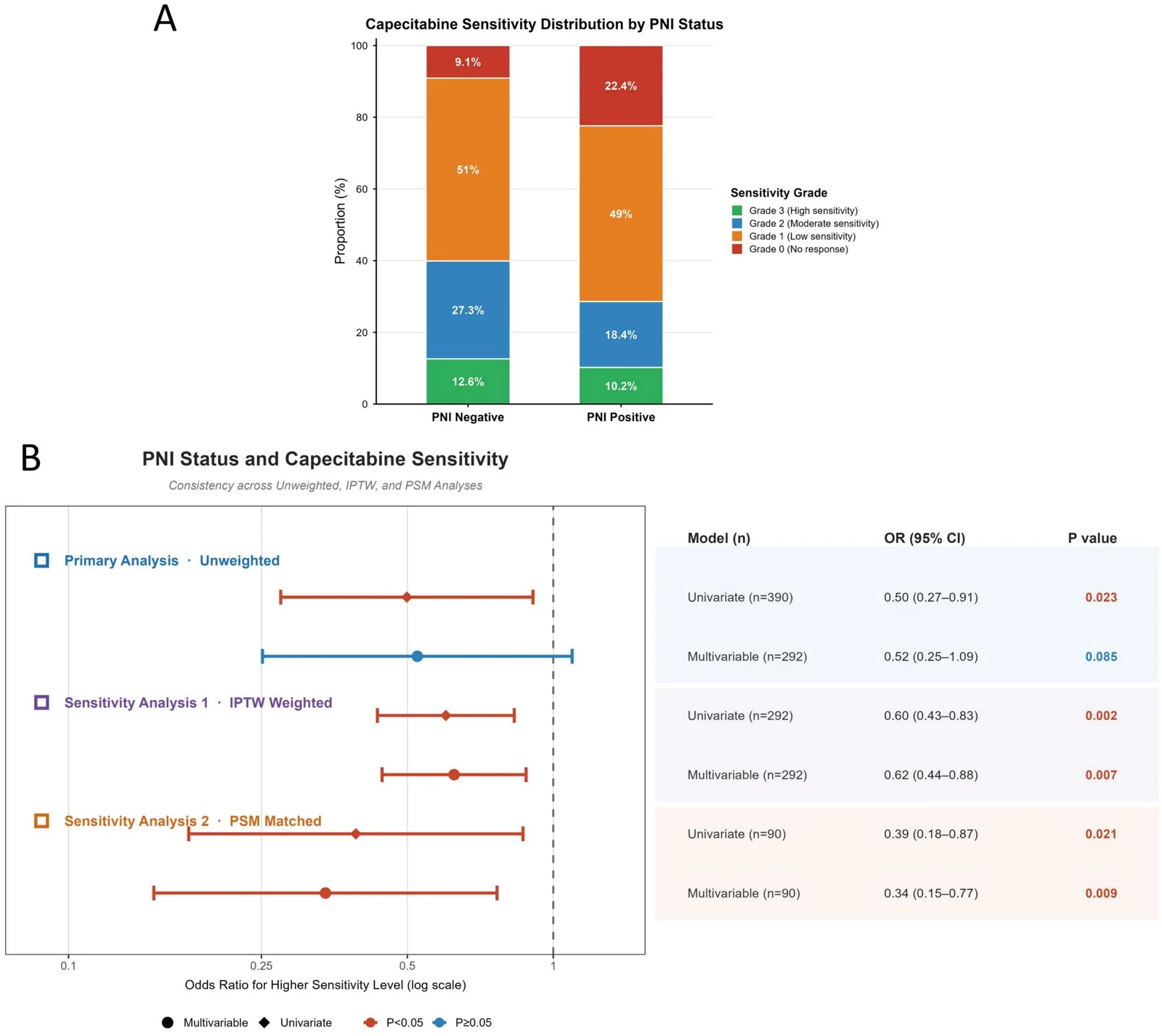
Association between perineural invasion status and *in vitro* capecitabine sensitivity. **(A)** Distribution of capecitabine sensitivity grades stratified by perineural invasion status, indicating a shift toward resistance in positive tumors. **(B)** Forest plot demonstrating consistent negative associations between perineural invasion and capecitabine sensitivity across unweighted, inverse probability of treatment weighting, and propensity score-matched analyses. PNI, perineural invasion; IPTW, inverse probability of treatment weighting; PSM, propensity score matching; OR, odds ratio; CI, confidence interval.

In the unweighted original cohort, univariate analysis indicated that PNI positivity was associated with reduced capecitabine sensitivity (OR = 0.50, 95% CI: 0.27–0.91), and the direction remained consistent after multivariable adjustment but did not reach statistical significance (95% CI 0.25–1.09, P = 0.085). To control for confounding, inverse probability of treatment weighting (IPTW; n = 292 after Ki-67 completeness restriction) was applied; covariate balance after weighting was satisfactory. PNI positivity was independently associated with reduced capecitabine sensitivity in both univariate (OR = 0.60, 95% CI: 0.43–0.83, P = 0.002) and multivariable (OR = 0.62, 95% CI: 0.44–0.88, P = 0.007) IPTW models. A 1:1 propensity score matching (PSM) robustness analysis (n = 90 matched pairs) yielded a univariate OR of 0.39 (95% CI: 0.18–0.87, P = 0.021) and a multivariable OR of 0.34 (95% CI: 0.15–0.77, P = 0.009); the wider confidence intervals reflect the reduced effective sample size after matching(**Figure 5B**). More detailed balance diagnostics were as follows. After IPTW, 11 of 13 covariates had ASMD < 0.1, while pT_2 and pT_3 showed mild residual imbalance with ASMD values of 0.135 and 0.180, respectively. The effective sample sizes were 246.5 for PNI-negative patients and 21.7 for PNI-positive patients. After PSM, all 11 covariates included in the matching model, namely pT, pN, LVI, tumor location, and KRAS, had ASMD = 0. The proportional odds assumption was formally tested. The Brant test did not indicate a significant violation in the PSM-matched multivariable model (global P = 0.77; PNI P = 0.83), the full Cohort B univariate model (global P = 0.32; PNI P = 0.32), or the complete-covariate multivariable model (global P = 0.42; PNI P = 0.31). A partial proportional odds model yielded directionally consistent results after inverting the coefficients (OR for higher capecitabine sensitivity: 0.26, 0.36, and 0.51; P = 0.035, 0.027, and 0.324). A multinomial logistic regression model also yielded consistent results (OR for Grades 1–3 vs Grade 0: 0.39, 0.19, 0.18). After controlling for measured confounders by IPTW and PSM, PNI was associated with reduced *in vitro* capecitabine sensitivity. However, residual confounding by unmeasured factors cannot be excluded.

## 4 Discussion

In this retrospective cohort study, we developed and validated a six-factor nomogram model for quantitatively assessing the risk of PNI in patients with CRC. The hybrid variable-selection approach may carry a risk of overfitting, but the calibration and decision curve analysis results support the final model. The model demonstrated acceptable discrimination and good calibration in both the training and independent validation cohorts. Furthermore, decision curve analysis indicated that the model provides a stable net clinical benefit within a clinically relevant threshold range. Beyond its utility for risk stratification, we also found that PNI-positive status was independently associated with distant metastasis and reduced *in vitro* sensitivity to capecitabine. These findings suggest the potential clinical utility of integrating PNI risk estimates into postoperative decision-making, pending prospective validation. Tumor location and pT stage showed imbalance between included and excluded patients, and complete-case analysis may introduce selection bias. However, the balance of PNI itself and the consistency of sensitivity analyses support the robustness of the primary finding.

The construction of this nomogram model is based on a series of widely recognized pathological indicators of tumor aggressiveness, including tumor invasion depth, nodal status, lymphatic vascular invasion (LVI), tumor budding, KRAS mutation status, and Ki-67 expression level. As the core framework for CRC risk assessment, the TNM staging system—specifically T stage and N stage—directly reflects the local invasive capacity and regional metastatic potential of the tumor. Tumor budding, defined as isolated single cells or clusters of up to four cells at the invasive front of colorectal carcinoma, represents a morphological manifestation of epithelial-mesenchymal transition (EMT). It has been established as a significant marker of an aggressive phenotype closely associated with advanced stage, LVI, and distant metastasis (14, 15). LVI represents a critical step in tumor cell entry into vascular or lymphatic channels and is a well-recognized adverse prognostic factor (16, 17). At the molecular level, this study identified a correlation between PNI and KRAS mutation status, suggesting that a subset of PNI-positive tumors may rely on specific driver pathways to facilitate local invasion (18). Existing evidence supports the independent value of KRAS status in predicting PNI, offering new insights into the role of KRAS in local tumor invasiveness (19). Ki-67 exhibited a negative coefficient in the multivariable model, and in our cohort the proportion of high Ki-67 expression was paradoxically lower in PNI-positive than in PNI-negative tumors (89% vs 96%, P = 0.013). A plausible interpretation is that PNI represents an invasion mode driven primarily by microenvironment-dependent neurotropic signaling rather than by rapid proliferation, such that PNI-positive tumors may preferentially adopt a more migratory, less proliferative phenotype; this hypothesis warrants confirmation by mechanistic studies. Therefore, the role of Ki-67 in the model should be interpreted as a covariate that adjusts for other related pathological features to enhance overall model performance, rather than serving as an independent mechanistic predictor of PNI (20). All these variables are routinely available in standard pathology workflows, enhancing the feasibility and reproducibility of the model in real-world clinical settings. Importantly, the clinical value of this model is not to replace direct pathological assessment of PNI. Instead, it serves as a supplementary tool for postoperative risk stratification and adjuvant therapy decision-making by providing an objective, quantitative PNI risk estimate when pathological evaluation is uncertain, subject to inter-observer variability, or limited by sampling constraints (20).

Consistent with previous studies, our findings reaffirm that PNI remained independently associated with distant metastasis in CRC (21). PNI reflects a highly aggressive tumor phenotype characterized by intimate interactions between tumor cells and the neural microenvironment, which may promote local invasion and facilitate systemic dissemination (22). In this study, this association remained robust across multiple clinicopathological subgroups, further underscoring the reliability of PNI as an aggressive biological marker. Subgroup analysis suggested a directionally stronger association in KRAS wild-type tumors, but the interaction test did not reach statistical significance and this finding should be considered exploratory. A potential biological explanation is that KRAS wild-type tumors may rely more heavily on microenvironment-driven neurotrophic signaling such as the BDNF/TrkB axis, whereas KRAS-mutant tumors already have constitutively activated endogenous oncogenic signaling, potentially reducing the additional effect of PNI. This hypothesis requires validation in larger prospective cohorts (3, 23). Notably, PNI did not demonstrate significant predictive value for metastasis in early-stage subgroups, such as T2 or N0/N1. This may be attributed to the immaturity of the nerve-tumor interaction network in early tumors or the likelihood that their invasive behavior is driven predominantly by neural-independent pathways (24). Of interest, recent studies have reported that PNI fails to predict outcomes in node-negative colon cancer, aligning with the observations from our subgroup analysis, although the underlying mechanisms require further investigation (25). Prior research has proposed that the biological impact of PNI may be closely linked to the depth of invasion. Superficial PNI involves the submucosal and myenteric plexuses, whereas deep PNI involves the subserosal plexus. Differences in invasive potential exist between “superficial” and “deep” PNI, providing a plausible explanation for the relatively weaker effect of PNI in early-stage tumors (26). In addition to the depth of invasion and the maturity of the nerve-tumor interaction network, emerging evidence suggests that the immunosuppressive tumor microenvironment may be a key determinant of whether PNI has prognostic significance in early-stage colorectal cancer. Tumor-associated macrophages (TAMs) are key regulators of PNI, and tumor-derived JAG2 activates NOTCH3 on macrophages, triggering STAT3-dependent CCL2 expression and reprogramming them toward an M2-like immunosuppressive phenotype (27). In node-negative early-stage tumors, this immunosuppressive program may not yet be fully established. Consistent with this hypothesis, S100-immunostained PNI was detected in 67.3% of stage I/II colorectal cancers and independently predicted worse overall mortality in early-stage but not late-stage disease, with a concomitant reduction in stromal lymphocytic reaction (28). Furthermore, PRRX2-driven MMP2-positive myofibroblastic cancer-associated fibroblasts (CAFs) mediate PNI through TGF-β/ECM remodeling, and their enrichment may be limited in node-negative cases (29). Collectively, the prognostic value of PNI depends on the immune and stromal composition of the tumor microenvironment, and future studies should integrate immune and stromal profiling with PNI depth assessment to refine risk stratification in early-stage colorectal cancer. Collectively, these results indicate that the prognostic value of PNI for predicting metastasis is not uniform across all patient populations but is modulated by a combination of factors, including tumor stage, molecular background, and depth of neural invasion.

Another significant finding of this study is the robust association between PNI-positive status and reduced *in vitro* sensitivity to capecitabine. This result remained consistent across multiple analytical frameworks, including multivariable adjustment, inverse probability of treatment weighting (IPTW), and propensity score matching (PSM). Although ATP-based *in vitro* chemosensitivity assays cannot fully recapitulate the complex pharmacokinetics and immune interactions *in vivo*, these findings suggest that PNI-positive tumors may harbor an intrinsic resistant phenotype to fluoropyrimidine-based chemotherapy. This discovery offers a potential biological explanation for why some PNI-positive patients derive limited benefit from standard adjuvant chemotherapy and supports the rationale for exploring treatment intensification or alternative therapeutic strategies in this population.

There is growing recognition that interactions between the nervous system and tumors may play a pivotal role in mediating chemotherapy resistance. Within the tumor microenvironment, neurons and glial cells not only remodel the extracellular matrix via paracrine factors—such as neurotrophic factors and neurotransmitters—but may also form synapse-like structures with tumor cells to directly modulate their biological behavior (30). Relevant studies have shown that PNI-positive patients often derive limited benefit from conventional chemotherapy, potentially due to neuro-associated signaling-mediated primary drug resistance (31). Further experimental research suggests that multiple neural signaling pathways related to apoptosis inhibition, epithelial-mesenchymal transition (EMT), metabolic reprogramming, and enhanced survival signals may be involved (32). For instance, the neurotrophic factor TrkB axis and adrenergic signaling have been reported to correlate with chemoresistance in CRC and other solid tumors (33, 34). Studies in animal models have further demonstrated that intervening in innervation or blocking nerve-related signaling can enhance the efficacy of various chemotherapeutic agents (35). Although targeted therapies directed at neural signaling remain limited in clinical application for CRC, these findings provide a rational foundation for exploring combinatorial strategies involving neural-targeted interventions alongside standard chemotherapy in patients exhibiting PNI and related phenotypic characteristics (36).

Several molecular biomarkers have been linked to PNI in colorectal cancer and may serve as candidates for future clinical translation. Tumor-associated Schwann cells play a pivotal role in PNI by promoting epithelial-mesenchymal transition, extracellular matrix remodeling, and immune modulation, and Schwann cell markers such as S100A4 have been found to be significantly associated with PNI in colorectal cancer. A recent bioinformatic analysis identified CLCF1 as a key prognostic marker associated with PNI, with elevated expression enhancing the proliferation, invasion, and migration of colorectal cancer cells (37). A prognostic nomogram incorporating PNI-associated genes including CLDN18 and FTCD has been developed for stage II colorectal cancer, achieving AUC values exceeding 0.7 for predicting 1-, 3-, and 5-year overall survival (38). These biomarkers may aid in identifying PNI-positive patients who could benefit from neurotrophic or adrenergic pathway inhibition combined with standard chemotherapy. Integrating such biomarkers with our six-factor nomogram could further refine postoperative risk stratification and guide individualized adjuvant therapy, although prospective validation is required.

This study has several limitations that should be acknowledged. First, the single-center retrospective design carries an inherent risk of selection bias and lacks an external, multicenter validation cohort. Second, the chemosensitivity analysis was based on *in vitro* experiments, which cannot fully capture the influence of host pharmacokinetics and immune factors *in vivo*. Capecitabine is a prodrug, and no exogenous activation system was added in this assay, so incomplete activation cannot be excluded. Future studies should validate these findings using 5-FU or active metabolites. And IPTW and PSM only control for measured covariates, and residual confounding by unmeasured factors cannot be excluded. Finally, owing to the limited follow-up, only five deaths were recorded in this cohort, precluding analysis of long-term survival endpoints such as disease-free survival (DFS) or overall survival (OS). Consequently, the predictive value of the model for long-term survival warrants further validation through prospective studies. Future investigations should employ prospective designs with extended follow-up periods and incorporate clinical efficacy outcomes to further verify the predictive utility and therapeutic implications of PNI-based risk stratification.

## 5 Conclusion

In summary, this study presents a practical six-factor nomogram based on routine pathological features for assessing the risk of postoperative PNI in patients with CRC. Furthermore, it reveals an independent association between PNI-positive status and enhanced metastatic potential as well as reduced *in vitro* sensitivity to capecitabine. These findings support the integration of PNI-related risk information into postoperative decision-making and lay a solid foundation for future exploration of individualized adjuvant therapeutic strategies for PNI-positive patients.

## Data Availability

The original contributions presented in the study are included in the article. Further inquiries can be directed to the corresponding author.

## References

[1] Siegel RL Kratzer TB Wagle NS Sung H Jemal A . Cancer statistics, 2026. Ca: A Cancer J For Clin. (2026) 76:e70043. doi: 10.3322/caac.70043 41528114 PMC12798275

[2] Liebig C Ayala G Wilks JA Berger DH Albo D . Perineural invasion in cancer: a review of the literature. Cancer. (2009) 115:3379–91. doi: 10.1002/cncr.24396 19484787

[3] Wang H Huo R He K Cheng L Zhang S Yu M . Perineural invasion in colorectal cancer: mechanisms of action and clinical relevance. Cell Oncol (Dordrecht Netherlands). (2024) 47:1–17. doi: 10.1007/s13402-023-00857-y 37610689 PMC10899381

[4] Que Y Wu R Li H Lu J . A prediction nomogram for perineural invasion in colorectal cancer patients: a retrospective study. BMC Surg. (2024) 24:80. doi: 10.1186/s12893-024-02364-9 38439014 PMC10913563

[5] Zheng Z Sun X . Development of a nomogram predicting perineural invasion risk and assessment of the prognostic value of perineural invasion in colon cancer: a population study based on the Surveillance, Epidemiology, and End Results database. Transl Cancer Res. (2025) 14:141–58. doi: 10.21037/tcr-24-1030 39974388 PMC11833362

[6] Zhang L Yang L Jiang S Yu M . Nerve dependence in colorectal cancer. Front Cell Dev Biol. (2022) 10:766653. doi: 10.3389/fcell.2022.766653 35223829 PMC8866866

[7] Wang H He K Huo R Li W Zhang S Jiang L . MAGEA6 engages a YY1-dependent transcription to dictate perineural invasion in colorectal cancer. Adv Sci (Weinheim Baden-Wurttemberg Germany). (2025) 12:e2501119. doi: 10.1002/advs.202501119 40145793 PMC12224987

[8] Wang Y Tan H Li S Long C Zhou B Wang Z . Dual-energy CT combined with histogram parameters in the assessment of perineural invasion in colorectal cancer. Int J Colorectal Dis. (2025) 40:129. doi: 10.1007/s00384-025-04919-5 40423818 PMC12116712

[9] Brouwer NPM Lord AC Terlizzo M Bateman AC West NP Goldin R . Interobserver variation in the classification of tumor deposits in rectal cancer-is the use of histopathological characteristics the way to go? Virchows Archiv Int J Pathol. (2021) 479:1111–18. doi: 10.1007/s00428-021-03197-0 34480612 PMC8724135

[10] Liebig C Ayala G Wilks J Verstovsek G Liu H Agarwal N . Perineural invasion is an independent predictor of outcome in colorectal cancer. J Clin Oncol Off J Am Soc Clin Oncol. (2009) 27:5131–37. doi: 10.1200/JCO.2009.22.4949 19738119 PMC2773472

[11] Twelves C Wong A Nowacki MP Abt M Burris HR Carrato A . Capecitabine as adjuvant treatment for stage III colon cancer. N Engl J Med. (2005) 352:2696–704. doi: 10.1056/NEJMoa043116 15987918

[12] Cassidy J Clarke S Díaz-Rubio E Scheithauer W Figer A Wong R . Randomized phase III study of capecitabine plus oxaliplatin compared with fluorouracil/folinic acid plus oxaliplatin as first-line therapy for metastatic colorectal cancer. J Clin Oncol Off J Am Soc Clin Oncol. (2008) 26:2006–12. doi: 10.1200/JCO.2007.14.9898 18421053

[13] Zahalka AH Frenette PS . Nerves in cancer. Nat Rev Cancer. (2020) 20:143–57. doi: 10.1038/s41568-019-0237-2 31974491 PMC7709871

[14] Lugli A Kirsch R Ajioka Y Bosman F Cathomas G Dawson H . Recommendations for reporting tumor budding in colorectal cancer based on the International Tumor Budding Consensus Conference (ITBCC) 2016. Mod Pathol Off J United States Can Acad Pathol Inc. (2017) 30:1299–311. doi: 10.1038/modpathol.2017.46 28548122

[15] Haddad TS Bokhorst J van den Dobbelsteen L Ztürk SK Baumann E van Vliet S . Tumor budding and poorly differentiated clusters as a biological continuum in colorectal cancer invasion and prognosis. Sci Rep. (2025) 15:16944. doi: 10.1038/s41598-025-00866-x 40374662 PMC12081641

[16] Al-Sukhni E Attwood K Gabriel EM LeVea CM Kanehira K Nurkin SJ . Lymphovascular and perineural invasion are associated with poor prognostic features and outcomes in colorectal cancer: a retrospective cohort study. Int J Surg (London England). (2017) 37:42–9. doi: 10.1016/j.ijsu.2016.08.528 27600906

[17] Yuan H Dong Q Zheng B Hu X Xu J Tu S . Lymphovascular invasion is a high risk factor for stage I/II colorectal cancer: a systematic review and meta-analysis. Oncotarget. (2017) 8:46565–79. doi: 10.18632/oncotarget.15425 28430621 PMC5542293

[18] Martianov AS Mitiushkina NV Ershova AN Martynenko DE Bubnov MG Amankwah P . KRAS, NRAS, BRAF, HER2 and MSI status in a large consecutive series of colorectal carcinomas. Int J Mol Sci. (2023) 24:4868. doi: 10.3390/ijms24054868 36902296 PMC10003572

[19] Chen K Collins G Wang H Toh JWT . Pathological features and prognostication in colorectal cancer. Curr Oncol (Toronto Ont). (2021) 28:5356–83. doi: 10.3390/curroncol28060447 34940086 PMC8700531

[20] Melling N Kowitz CM Simon R Bokemeyer C Terracciano L Sauter G . High Ki67 expression is an independent good prognostic marker in colorectal cancer. J Clin Pathol. (2016) 69:209–14. doi: 10.1136/jclinpath-2015-202985 26281861

[21] Yang Y Huang X Sun J Gao P Song Y Chen X . Prognostic value of perineural invasion in colorectal cancer: a meta-analysis. J Gastrointestinal Surg Off J Soc For Surg Aliment Tract. (2015) 19:1113–22. doi: 10.1007/s11605-015-2761-z 25663635

[22] Ebrahim NAA Soliman SMA Othman MO Tahoun NS . Molecular mechanisms and clinical significance of perineural invasion in malignancies: the pivotal role of tumor-associated Schwann cells in cancer progression and metastasis. Med Oncol (Northwood London England). (2025) 42:171. doi: 10.1007/s12032-025-02729-x 40259163

[23] Tanaka K Okugawa Y Toiyama Y Inoue Y Saigusa S Kawamura M . Brain-derived neurotrophic factor (BDNF)-induced tropomyosin-related kinase B (Trk B) signaling is a potential therapeutic target for peritoneal carcinomatosis arising from colorectal cancer. PLoS One. (2014) 9:e96410. doi: 10.1371/journal.pone.0096410 24801982 PMC4011754

[24] Zhao J Cheng L Yang J Xu F Qi W Liao K . Tumor-nerve interactions in cancer regulation and progression. Cancer Lett. (2025) 612:217483. doi: 10.1016/j.canlet.2025.217483 39842496

[25] Chu C Lai I Jong B Chiang S Tsai W Hsieh P . The prognostic and predictive significance of perineural invasion in stage I to III colon cancer: a propensity score matching-based analysis. World J Surg Oncol. (2024) 22:129. doi: 10.1186/s12957-024-03405-6 38734718 PMC11088143

[26] Chen H Wang C Chen Z Huang T Lin Y Chen J . The depth of perineural invasion is an independent prognostic factor for stage II colorectal cancer. BMC Cancer. (2024) 24:433. doi: 10.1186/s12885-024-12206-9 38589842 PMC11003015

[27] Zhao E Liu R Chen N Zhou C Lin D Liang X . Tumor-derived JAG2 programs macrophages via NOTCH3 to drive perineural invasion in colorectal cancer. Int J Biol Macromol. (2026) 358:151724. doi: 10.1016/j.ijbiomac.2026.151724 41916520

[28] Fukuda Y Tanaka Y Eto K Ukai N Sonobe S Takahashi H . S100-stained perineural invasion is associated with worse prognosis in stage I/II colorectal cancer: its possible association with immunosuppression in the tumor. Pathol Int. (2022) 72:117–27. doi: 10.1111/pin.13195 35007380

[29] Chen M Cai Y Han F Li B Xu Z Cui K . Single-cell and bulk RNA-sequencing reveal PRRX2-driven cancer-associated fibroblast-mediated perineural invasion for predicting the immunotherapy outcome in colorectal cancer. Front Cell Dev Biol. (2025) 13:1620388. doi: 10.3389/fcell.2025.1620388 41089673 PMC12516196

[30] Mancusi R Monje M . The neuroscience of cancer. Nature. (2023) 618:467–79. doi: 10.1038/s41586-023-05968-y 37316719 PMC11146751

[31] Li J Mei S Zhou S Zhao F Liu Q . Perineural invasion is a prognostic factor in stage II colorectal cancer but not a treatment indicator for traditional chemotherapy: a retrospective cohort study. J Gastrointest Oncol. (2022) 13:710–21. doi: 10.21037/jgo-22-277 35557585 PMC9086063

[32] Varghese E Samuel SM Hornak S Kubatka P Büsselberg D . Unraveling the nexus in the neuro-neoplastic progression of colorectal cancer: potential role of β2-adrenergic receptor (β2-AR). J Adv Res. (2026) 83:1005–19. doi: 10.1016/j.jare.2025.08.052 40914425 PMC13131394

[33] Zeng Z Cai S Ye C Li T Tian Y Liu E . Neural influences in colorectal cancer progression and therapeutic strategies. Int J Colorectal Dis. (2025) 40:120. doi: 10.1007/s00384-025-04887-w 40379990 PMC12084286

[34] Komine C Sohda M Yokobori T Shioi I Ozawa N Shibasaki Y . Impact of tumoral β2-adrenergic receptor expression on chemotherapeutic response and prognosis in patients with advanced colorectal cancer. Ann Surg Oncol. (2025) 32:1913–24. doi: 10.1245/s10434-024-16195-8 39341920

[35] Manoleras AV Sloan EK Chang A . The sympathetic nervous system shapes the tumor microenvironment to impair chemotherapy response. Front Oncol. (2024) 14:1460493. doi: 10.3389/fonc.2024.1460493 39381049 PMC11458372

[36] Gouda MA Thein KZ Hong DS . Tissue-agnostic targeting of neurotrophic tyrosine receptor kinase fusions: current approvals and future directions. Cancers (Basel). (2024) 16:3395. doi: 10.3390/cancers16193395 39410015 PMC11475940

[37] Zhang W Gao Y Zhang N Su J Li S Wang Y . CLCF1 is associated with perineurial invasion and prognosis in colorectal cancer. Sci Rep. (2025) 15:39747. doi: 10.1038/s41598-025-15358-1 41233365 PMC12615840

[38] Chang C Zhang B Chen J Wang G . Identification of key genes associated with perineural invasion in stage II colorectal cancer and their prognostic implications. Front Oncol. (2025) 15:1612143. doi: 10.3389/fonc.2025.1612143 40936686 PMC12420321

